# 284 nm UVB Phototherapy Regulates Immunoinflammatory Responses and Improves Atherosclerotic Plaque Stability in Mice

**DOI:** 10.24546/0100497749

**Published:** 2025-10-03

**Authors:** KEN ITO, NAOTO SASAKI, AGA KRISNANDA, TORU TANAKA, SAYO HORIBE, MOTOAKI IWAYA, ATSUSHI FUKUNAGA, YOSHIYUKI RIKITAKE

**Affiliations:** 1 Laboratory of Medical Pharmaceutics, Kobe Pharmaceutical University, Kobe, Japan; 2 Division of Cardiovascular Medicine, Department of Internal Medicine, Kobe University Graduate School of Medicine, Kobe, Japan; 3 Department of Materials Science and Engineering, Meijo University, Nagoya, Japan; 4 Department of Dermatology, Division of Medicine for Function and Morphology of Sensory Organs, Faculty of Medicine, Osaka Medical and Pharmaceutical University, Osaka, Japan

**Keywords:** Atherosclerosis, Plaque stabilization, Ultraviolet B, T cells, Inflammation

## Abstract

**AIM:**

We previously reported that broad-band and specific wavelengths of ultraviolet B (UVB) limit the development of atherosclerosis by augmenting anti-inflammatory immune responses in hypercholesterolemic mice. This study aimed to elucidate the effect of 284 nm UVB on the stabilization of atherosclerotic plaques and the underlying mechanisms.

**METHODS AND RESULTS:**

Six- to eight-week-old male LDL receptor-deficient (*Ldlr^−/−^*) mice were fed a high-fat and high-cholesterol diet for 8 weeks to form atherosclerotic lesions, and the diet was changed to a standard diet. The mice were subsequently irradiated with 284 nm UVB at 5 kJ/m^2^ twice weekly for 2 or 4 weeks. The effects of 284 nm UVB irradiation on atherosclerotic plaque size and components and immunoinflammatory responses were evaluated by histological analysis and flow cytometry. Normalization of plasma cholesterol levels did not prevent the development of atherosclerotic lesions in the aortic sinus of UVB-irradiated or nonirradiated mice, whereas it markedly reduced the lipid content in the aortic sinus lesions of these mice, which was more prominent in UVB-irradiated mice. The accumulation of CD4^+^ T cells in atherosclerotic lesions and aortic immunoinflammatory responses were reduced in UVB-irradiated mice, although no beneficial effects of UVB treatment on macrophage accumulation or collagen content were observed. The plaque-stabilizing effect of UVB treatment was associated with augmented regulatory T cell immune responses in lymphoid tissues.

**CONCLUSIONS:**

284 nm UVB irradiation in combination with lipid-lowering therapy improves the stability of atherosclerotic plaques by augmenting anti-inflammatory Treg immune responses. The combination of aggressive lipid-lowering therapies and 284 nm UVB phototherapy may serve as an attractive therapeutic approach for high-risk patients with vulnerable atherosclerotic plaques.

## INTRODUCTION

Atherosclerosis induces coronary artery disease (CAD) and stroke, which are major causes of mortality worldwide. Firm evidence indicates that chronic aortic inflammation is responsible for the initiation and progression of atherosclerotic disease (1). Although advanced medical treatment can partially reduce the risk of atherosclerotic disease, patients with this disease still have considerable residual risk related to chronic inflammation (2). Emerging clinical evidence suggests that anti-inflammatory therapies in combination with the regulation of CAD risk factors, including intensive lipid-lowering therapy, are effective for preventing CAD in patients with a past disease history (3, 4).

The involvement of dysregulation of innate and adaptive immunity in aortic inflammation is no longer controversial (1). Activated and differentiated T cells are dominant among the infiltrated immune cells in human atherosclerotic plaques, indicating a possible role of T cells in the development of atherosclerosis (5). A large number of experimental studies support the findings of human studies, although there are some differences in the immune system between mice and humans. CD4^+^ effector T cell (Teff) subsets are divided into T helper type 1 (Th1), T helper type 2 (Th2), and T helper type 17 (Th17) cells, which have diverse and complex roles in atherosclerosis (6). Among these subsets, Th1 cells play a proatherogenic role by producing the proinflammatory cytokine interferon (IFN)-γ (1). Forkhead box P3 (Foxp3)-expressing regulatory T cells (Tregs) are indispensable for regulating pathogenic immunoinflammatory responses and autoimmune disorders and have been shown to protect against atherosclerosis (7). Clinical data also suggest the possible atheroprotective role of Tregs in humans (8). Several experimental approaches including the administration of antibodies (9, 10), cytokines (11), and an active form of vitamin D_3_ (12) modulate the Teff/Treg balance toward Treg responses and effectively limit the development of atherosclerosis. We previously reported that intravenous injection of anti-CD3 antibodies regressed established atherosclerotic lesions and improved their stability by increasing the proportion of Tregs in lymphoid tissues and atherosclerotic lesions (10). In support of our findings, an experimental study showed the accumulation of Tregs in regressing plaques, which may play a critical role in promoting inflammation resolution in the aorta (13). These reports suggest that Tregs play a critical role in the regulation of both early and advanced atherosclerotic lesions.

Based on the anti-inflammatory actions of ultraviolet B (UVB), UVB phototherapy is an established treatment for immunoinflammatory cutaneous diseases (14). We recently reported that irradiation with broad-band UVB (a continuous spectrum from 280 to 320 nm with a peak at approximately 313 nm), 282–284 nm UVB (a narrow peak at approximately 282–284 nm), or 312 nm UVB (a narrow peak at approximately 312 nm) inhibits atherosclerotic plaque formation by augmenting anti-inflammatory Treg immune responses in hypercholesterolemic apolipoprotein E-deficient (*Apoe*^−/−^) mice (15–17), providing an attractive approach for preventing atherosclerosis. Based on these reports, we found that 282–284 nm UVB has a potent anti-atherogenic effect compared with broad-band or 312 nm UVB. However, the effect of 282–284 nm UVB treatment on the stabilization of advanced atherosclerotic lesions has not been investigated. The elucidation of its protective effect could lead to the development of new therapeutic approaches to improve plaque stability and reduce cardiovascular events.

In this study, using a previously established mouse model of atherosclerosis (18), we investigated the effect of the combination of diet-induced lipid-lowering therapy and 284 nm UVB irradiation on the stabilization of established atherosclerotic lesions and the underlying mechanisms, focusing on immunoinflammatory responses including T cell immune responses.

## MATERIALS AND METHODS

### Animals and experimental design

LDL receptor-deficient (*Ldlr*^−/−^) mice on a C57BL/6 background were previously described (19). Our mouse model of atherosclerosis was previously described (10, 18). Six- to eight-week-old male *Ldlr*^−/−^ mice were fed a high-fat and high-cholesterol diet (21% fat, 1.25% cholesterol, and 0.5% cholate; Oriental Yeast, Tokyo, Japan) for 8 weeks to form atherosclerotic lesions. At 14–16 weeks of age, the diet was changed to a standard diet (CLEA, Tokyo, Japan) and continued until euthanasia. Mice were irradiated with 284 nm UVB twice weekly for 2 or 4 weeks and euthanized for the analysis of atherosclerotic lesions or immune responses. Just before the diet change, mice were euthanized as the baseline group. Nonirradiated mice served as controls. Mice were housed in cages for each treatment group in a specific pathogen-free animal facility at Kobe Pharmaceutical University. All animal experiments were approved and registered by the Animal Care Committee of Kobe Pharmaceutical University (Permit Numbers: 2023-036, 2024-012, and 2025-052) and conformed to the National Institutes of Health Guide for the Care and Use of Laboratory Animals and the ARRIVE guidelines (Animal Research: Reporting of *In Vivo* Experiments).

### UVB irradiation

A light-emitting diode (LED) lamp (Nikkiso Co., Ltd., Japan) that can emit 284 nm a wavelength of UVB was previously developed (16) and used in this study. The irradiance of the 284 nm UVB was 5.88 J/m^2^/second at a distance of 14 cm. Mice were placed 14 cm below the bank of lamps and irradiated for the indicated weeks after shaving their backs in the animal facility. Nonirradiated mice were not subjected to these procedures. The UVB irradiation dose used in this study was 5 kJ/m^2^. Randomization and allocation concealment were performed. Littermate mice were equally allocated to each treatment group. During the experiments, animal/cage location was not controlled. Investigators were not blinded to treatment allocation. Criterion for exclusion was defined as serious burns, but during at least 3 observations per week we did not observe such symptoms, and no UVB-irradiated mice were excluded. UVB irradiation was performed in our animal facility, and other experimental procedures were performed in our laboratory rooms.

### Assessment of biochemical parameters

After overnight fasting, blood was collected via cardiac puncture under anesthesia by intraperitoneal injection of medetomidine hydrochloride (0.3 mg/kg), midazolam (4 mg/kg), and butorphanol tartrate (5 mg/kg) (all WAKO). Plasma was obtained via centrifugation and stored at −80°C until measurement. The concentrations of plasma total cholesterol, high-density lipoprotein-cholesterol, and triglyceride were determined enzymatically using an automated chemistry analyzer (Oriental Yeast Co., Ltd., Tokyo, Japan).

### Assessment of atherosclerotic lesions

Atherosclerotic plaques in the aortic sinus or thoracoabdominal aorta, including the aortic arch, are typically examined in murine models of atherosclerosis. In this study, atherosclerotic plaques in the aortic sinus were evaluated for the following reasons. The aortic sinus consistently exhibits plaque formation on the interior vascular wall near the aortic valve, and the aortic valve itself serves as a landmark, making sample collection conditions easy to standardize. Additionally, the plaques formed in this region are usually larger than those formed at other sites, facilitating a more precise quantitative assessment of their detailed characteristics. Mice were anesthetized as described above, and the aorta was perfused with saline. The aorta was dissected from the middle of the left ventricle to the bifurcation of the iliac artery. For aortic root lesion analysis, samples were cut from the ascending aorta, and proximal samples containing the aortic sinus were embedded in OCT compounds (Tissue-Tek; Sakura Finetek, Tokyo, Japan). Five consecutive sections (10 μm thickness), spanning 600 μm of the aortic sinus, were collected from each mouse and stained with hematoxylin-eosin or Oil Red O (WAKO). Stained sections were digitally captured using a fluorescence microscope (BZ-X810; KEYENCE, Osaka, Japan). For quantitative analysis of atherosclerosis, the total lesion area of 5 separate sections from each mouse was obtained using the ImageJ (National Institutes of Health).

### Histological analysis of atherosclerotic lesions

Immunohistochemistry was performed on 4% paraformaldehyde-fixed cryosections (10 μm) of mouse aortic roots using antibodies to identify macrophages (MOMA-2, 1:400; BMA Biomedicals) and T cells (CD4, 1:100; BD Biosciences), followed by detection with biotinylated secondary antibodies and streptavidin-horseradish peroxidase. Masson’s trichrome staining was performed to delineate the fibrous area. Stained sections were digitally captured using a fluorescence microscope (BZ-810; KEYENCE), and the percentage of the stained area (the stained area per total atherosclerotic lesion area) was calculated. The quantification of CD4^+^ T cells was performed by counting positively stained cells, divided by the total plaque area.

### Flow cytometry

For flow cytometric analysis of lymphoid tissues, skin-draining lymph node (LN) cells and splenocytes were isolated and stained in PBS containing 2% fetal calf serum, as described previously (16). We used axillary and inguinal LNs as skin-draining LNs. Intracellular staining of Foxp3 was performed using a Foxp3 staining buffer set (Thermo Fisher Scientific) according to the manufacturer’s instructions. In some experiments, splenocytes were stimulated with 20 ng/ml phorbol 12-myristate 13-acetate (Sigma) and 1 mmol/L ionomycin (Sigma) for 5 hours in the presence of Brefeldin A (Thermo Fisher Scientific), and intracellular cytokine staining was performed as described previously (16). Flow cytometric analysis was performed using FACSAria III (BD Biosciences) with FlowJo software version 10.10.0 (Tree Star). The antibodies used are listed in Table I.

### Preparation of peritoneal macrophages

Six- to eight-week-old male *Ldlr*^−/−^ mice were fed a high-fat and high-cholesterol diet for 8 weeks, after which the diet was changed to a standard diet. Some mice were irradiated with 284 nm UVB twice weekly for 4 weeks. UVB-irradiated and nonirradiated mice were intraperitoneally injected with 3% thioglycollate broth (Sigma) and euthanized for peritoneal macrophage isolation at 3 days after the injection. Cells were plated onto culture dishes with RPMI 1640 medium (Sigma) supplemented with 10% fetal calf serum and incubated at 37°C with 5% CO_2_ for 2 hours. Adhesive cells were used as macrophages for real-time reverse transcription PCR.

### Real-time reverse transcription PCR

Total RNA was extracted from the thoracoabdominal aorta or from peritoneal macrophages using TRIzol reagent (Life Technologies). For reverse transcription, a PrimeScript RT reagent Kit (Takara) was used. Quantitative PCR was performed using a TB Green Premix Ex Taq (Takara) and a StepOnePlus Real-Time PCR System (Thermo Fisher Scientific) according to the manufacturer’s protocol. The primers used are listed in Table II.

### Statistical analysis

Normality was assessed by Shapiro-Wilk normality test. Mann-Whitney *U*-test or 2-tailed Student’s or Welch’s *t*-test was used to detect significant differences between 2 groups where appropriate. One-way ANOVA followed by Tukey’s multiple comparisons test, or Kruskal-Wallis test followed by Dunn’s post hoc test was performed where appropriate to compare 3 groups comprising the baseline and mice at each time point. A value of *P* < 0.05 was considered statistically significant. Data are expressed as means ± s.d. No data were excluded from the analysis. The investigators were not blinded to the data analysis. For statistical analysis, GraphPad Prism version 8.0 (GraphPad Software Inc.) was used.

## RESULTS

### Normalization of plasma cholesterol levels and 284 nm UVB irradiation reduce the lipid content of atherosclerotic plaques

**Fig. 1:**
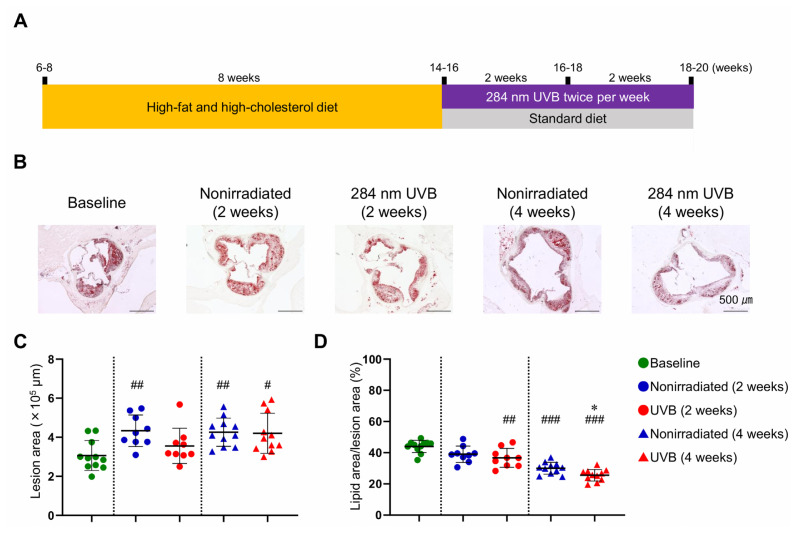
Normalization of plasma cholesterol levels and 284 nm ultraviolet B (UVB) irradiation reduce the lipid content of atherosclerotic plaques Six- to eight-week-old male LDL receptor-deficient (*Ldlr*^−/−^) mice were fed a high-fat and high-cholesterol diet for 8 weeks to induce atherosclerotic lesions. At 14–16 weeks of age, the diet was changed to a standard diet, and the mice were irradiated with 284 nm UVB at 5 kJ/m^2^ twice weekly for 2 or 4 weeks. Atherosclerotic lesions were analyzed at 14–16 (baseline), 16–18, and 18–20 weeks of age. Nonirradiated male *Ldlr*^−/−^ mice served as controls. **A**, Experimental design. **B**, Representative photomicrographs of Oil Red O staining in the aortic sinus. **C** and **D**, Quantitative analysis of atherosclerotic lesion area (**C**) and Oil Red O-stained positive area (lipid content) (**D**) in the aortic sinus. n = 9 to 11 per group. Black bars represent 500 μm as described. Data points represent individual animals. Horizontal bars represent means. Error bars indicate s.d. ^#^*P* < 0.05, ^##^*P* < 0.01, ^###^*P* < 0.001 vs. baseline; Kruskal-Wallis test followed by Dunn’s post hoc test: **C**; 1-way ANOVA followed by Tukey’s multiple comparisons test: **D**. *P < 0.05 vs. nonirradiated mice (4 weeks); 1-way ANOVA followed by Tukey’s multiple comparisons test.

We used a mouse model of atherosclerosis that was established in our previous studies (10, 18), and the experimental design is described in Fig. 1A. Six- to eight-week-old male *Ldlr*^−/−^ mice were fed a high-fat and high-cholesterol diet for 8 weeks to induce atherosclerotic lesions in the aortic sinus. At 14–16 weeks of age, the diet was changed to a standard diet, and the mice were irradiated with 284 nm UVB twice weekly for 2 or 4 weeks. No detrimental effects of UVB treatment, including skin cancer and burns, were observed throughout the observation. There was no difference in body weight between UVB-irradiated and nonirradiated mice (Table III). The change in the diet dramatically decreased the plasma total cholesterol levels in both groups compared with the baseline (Table III). No significant difference was observed in plasma total cholesterol levels between the 2 groups (Table III). To evaluate whether stabilization of atherosclerotic lesions was achieved, the lesion area in the aortic sinus was assessed at 2 or 4 weeks after the diet change in each group. A significant increase in the plaque area was observed in UVB-irradiated mice at 4 weeks and nonirradiated mice at 2 and 4 weeks after the diet change (Fig. 1B and 1C). The lipid content of established plaques was significantly decreased in UVB-irradiated mice at 2 and 4 weeks and nonirradiated mice at 4 weeks after the diet change (Fig. 1B and 1D). The plaque area was not significantly changed, and the lipid content was markedly decreased in UVB-irradiated mice at 2 weeks after the diet change (Fig. 1D), indicating the atheroprotective effect of UVB treatment at this time point. Means ± s.d. of lipid content proportion in the aortic sinus atherosclerotic lesions was 44.0 ± 3.9% at baseline, 39.0 ± 5.3% in nonirradiated mice at 2 weeks after the diet change, 36.7 ± 6.1% in UVB-irradiated mice at 2 weeks after the diet change, 30.0 ± 3.8% in nonirradiated mice at 4 weeks after the diet change, and 25.5 ± 3.7% in UVB-irradiated mice at 4 weeks after the diet change, as shown in Fig. 1D. Notably, the lipid content in the atherosclerotic lesions was significantly lower in UVB-irradiated mice than in nonirradiated mice at 4 weeks after the diet change (Fig. 1D), indicating the plaque-stabilizing effect of UVB treatment in combination with lipid-lowering therapy.

### Normalization of plasma cholesterol reduces the accumulation of inflammatory cells and increases collagen content in plaques, which is partially promoted by 284 nm UVB irradiation

**Fig. 2:**
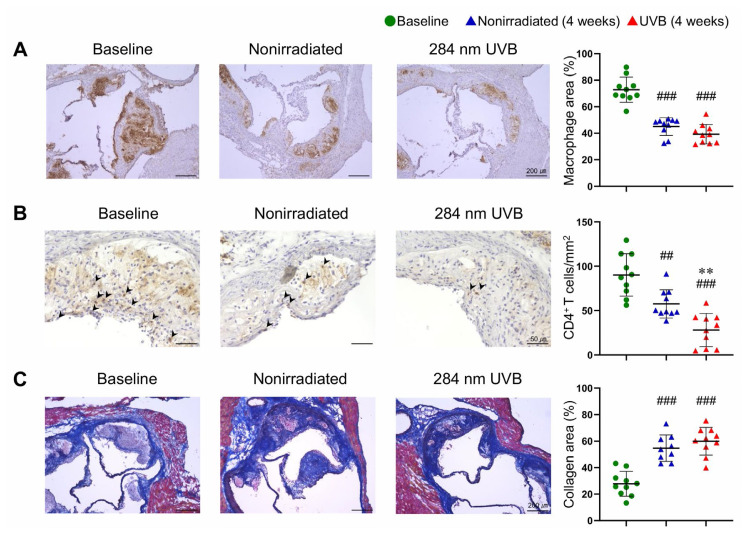
Normalization of plasma cholesterol reduces the accumulation of inflammatory cells and increases collagen content in plaques, which is partially promoted by 284 nm ultraviolet B (UVB) irradiation Six- to eight-week-old male LDL receptor-deficient (*Ldlr*^−/−^) mice were fed a high-fat and high-cholesterol diet for 8 weeks to induce atherosclerotic lesions. At 14–16 weeks of age, the diet was changed to a standard diet, and the mice were irradiated with 284 nm UVB at 5 kJ/m^2^ twice weekly for 4 weeks. Atherosclerotic lesions were analyzed at 14–16 (baseline) and 18–20 weeks of age. Nonirradiated male *Ldlr*^−/−^ mice served as controls. **A**–**C**, Representative sections and quantitative analyses of MOMA-2^+^ macrophages (**A**), CD4^+^ T cells (**B**), and collagen (**C**) in the aortic sinus. Black arrowheads indicate CD4^+^ T cells. n = 10 per group. Black bars represent 50 or 200 μm as described. Data points represent individual animals. Horizontal bars represent means. Error bars indicate s.d. ^##^*P* < 0.01, ^###^*P* < 0.001 vs. baseline; 1-way ANOVA followed by Tukey’s multiple comparisons test. **P < 0.01 vs. nonirradiated mice (4 weeks); 1-way ANOVA followed by Tukey’s multiple comparisons test.

To determine whether lipid-lowering therapy and UVB irradiation affect atherosclerotic plaque components, we analyzed atherosclerotic lesions in the aortic sinus by immunohistochemistry. Expectedly, normalization of plasma cholesterol significantly decreased the accumulation of macrophages and CD4^+^ T cells in the atherosclerotic lesions of UVB-irradiated and nonirradiated mice (Fig. 2A and 2B). The accumulation of CD4^+^ T cells in atherosclerotic lesions was lower in UVB-irradiated mice than in nonirradiated mice (Fig. 2B), whereas UVB irradiation had no beneficial effects on macrophage infiltration in the lesions (Fig. 2A). We next evaluated collagen fibers in the aortic sinus lesions using Masson’s trichrome staining. Normalization of plasma cholesterol dramatically increased the proportion of collagen content in the atherosclerotic lesions of UVB-irradiated and nonirradiated mice, whereas there was no difference in the composition between the 2 groups (Fig. 2C).

These data suggest that normalization of plasma cholesterol levels via diet modification substantially contributes to the stabilization of established plaques, which is partially promoted by UVB irradiation.

### 284 nm UVB irradiation augments Treg immune responses in the peripheral lymphoid tissues of mice with stabilized plaques

**Fig. 3:**
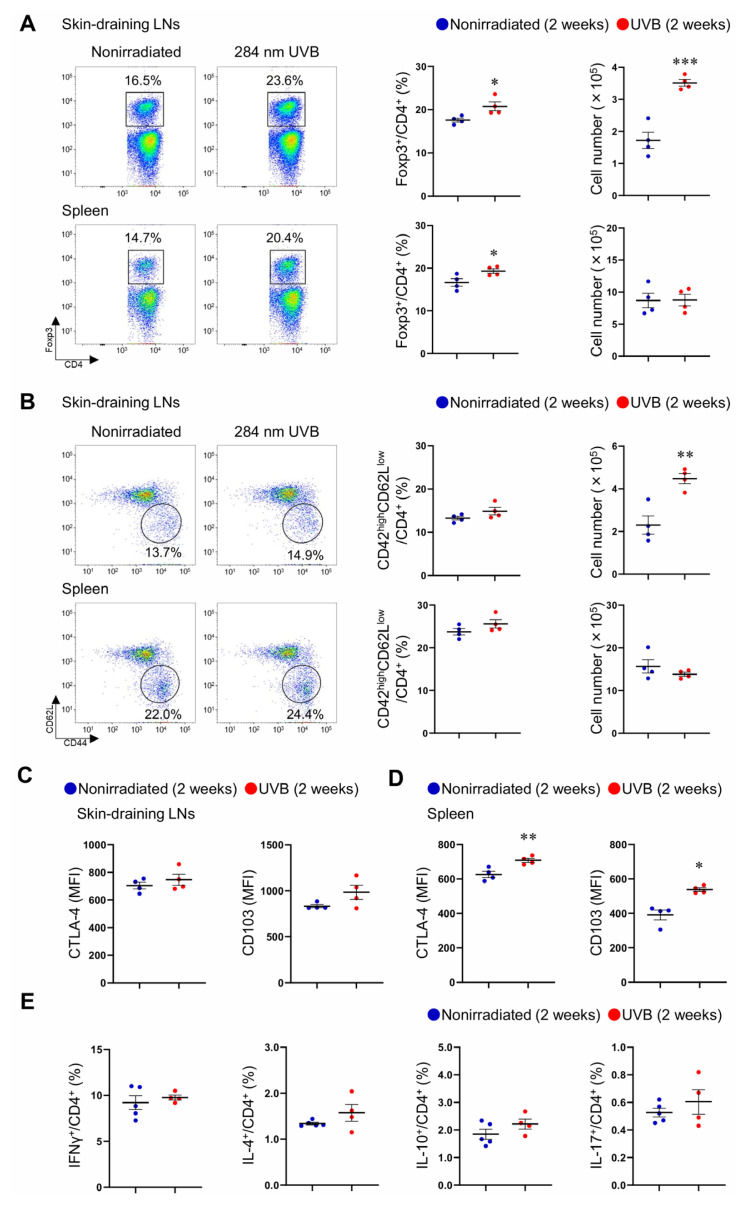
284 nm ultraviolet B (UVB) irradiation augments Treg immune responses in the peripheral lymphoid tissues of mice with stabilized plaques Six- to eight-week-old male LDL receptor-deficient (*Ldlr*^−/−^) mice were fed a high-fat and high-cholesterol diet for 8 weeks to induce atherosclerotic lesions. At 14–16 weeks of age, the diet was changed to a standard diet, and the mice were irradiated with 284 nm UVB at 5 kJ/m^2^ twice weekly for 2 weeks. Four days after the last UVB irradiation, lymphoid cells from the skin-draining lymph nodes (LNs) and spleen were collected. Nonirradiated male *Ldlr*^−/−^ mice served as controls. **A** and **B**, Representative flow cytometric analysis of CD4^+^ forkhead box P3 (Foxp3)^+^ Tregs (**A**) and CD4^+^CD44^high^CD62L^low^ effector memory T cells (**B**) in the skin-draining LNs and spleen. The graphs represent the proportions and total numbers of CD4^+^Foxp3^+^ Tregs (**A**) and CD4^+^CD44^high^CD62L^low^ effector memory T cells (**B**) in skin-draining LNs and spleen. n = 4 per group. **C** and **D**, The expression levels of cytotoxic T lymphocyte-associated antigen-4 (CTLA-4) and CD103 were analyzed by gating on CD4^+^Foxp3^+^ Tregs in the skin-draining LNs (**C**) and spleen (**D**). n = 4 per group. **E**, Lymphoid cells from spleen were stimulated with phorbol 12-myristate 13-acetate and ionomycin *in vitro*. Intracellular cytokine staining was performed. The graphs represent the frequencies of interferon (IFN)-γ^+^, interleukin (IL)-4^+^, IL-10^+^, and IL-17^+^ CD4^+^ T cells in spleen. n = 4 to 5 per group. Data points represent individual animals. Horizontal bars represent means. Error bars indicate s.d. *P < 0.05, **P < 0.01, ***P < 0.001; Mann-Whitney *U*-test: **D** right; 2-tailed Student’s *t*-test: **A**, **B**, and **D** left. MFI indicates mean fluorescence intensity.

To elucidate the mechanisms by which UVB irradiation improves the stability of atherosclerotic lesions, we focused on the immune responses of CD4^+^ T cells, including CD4^+^Foxp3^+^ Tregs, in peripheral lymphoid tissues. *Ldlr*^−/−^ mice were fed a high-fat and high-cholesterol diet for 8 weeks, and the diet was changed to a standard diet. We subsequently irradiated the mice with 284 nm UVB twice weekly for 2 weeks and performed flow cytometric analysis of the populations of CD4^+^Foxp3^+^ Tregs and CD4^+^CD44^high^CD62L^low^ effector memory T cells in the skin-draining LNs and spleen. 284 nm UVB irradiation significantly increased the frequency and number of CD4^+^Foxp3^+^ Tregs in the skin-draining LNs of *Ldlr*^−/−^ mice under the conditions of plasma cholesterol normalization (Fig. 3A). The frequency of CD4^+^Foxp3^+^ Tregs was also increased in the spleen of 284 nm UVB-irradiated mice, whereas the absolute cell number was not changed (Fig. 3A). 284 nm UVB irradiation had no major effect on the frequency and number of CD4^+^CD44^high^CD62L^low^ effector memory T cells in peripheral lymphoid tissues, although the absolute cell number was significantly increased in the skin-draining LNs of 284 nm UVB-irradiated mice (Fig. 3B).

To determine the effect of 284 nm UVB on the functionality and activation state of CD4^+^Foxp3^+^ Tregs, we performed flow cytometric analysis of activation- and function-associated molecules in CD4^+^Foxp3^+^ Tregs in the skin-draining LNs and spleen. Although the expression of cytotoxic T lymphocyte-associated antigen-4 (CTLA-4) and CD103 was not altered in CD4^+^Foxp3^+^ Tregs in the skin-draining LNs of UVB-irradiated mice (Fig. 3C), it was markedly upregulated in splenic CD4^+^Foxp3^+^ Tregs (Fig. 3D), implying the augmented suppressive function of systemically expanded Tregs. We investigated the effect of 284 nm UVB on CD4^+^ T cell subsets using intracellular cytokine staining. The frequencies of proatherogenic IFN-γ-producing Th1 cells, IL-4-producing Th2 cells, atheroprotective IL-10-producing CD4^+^ T cells, and IL-17-producing Th17 cells in spleen were not different between UVB-irradiated and nonirradiated mice (Fig. 3E).

Collectively, these data indicate that immunomodulation by 284 nm UVB irradiation can favorably shift the Teff/Treg balance toward Treg responses and may be specific to Treg responses, excluding the possibility of unwanted general immunosuppression.

### 284 nm UVB irradiation regulates aortic immunoinflammatory responses and induces a pro-resolving macrophage phenotype in mice with stabilized plaques

**Fig. 4:**
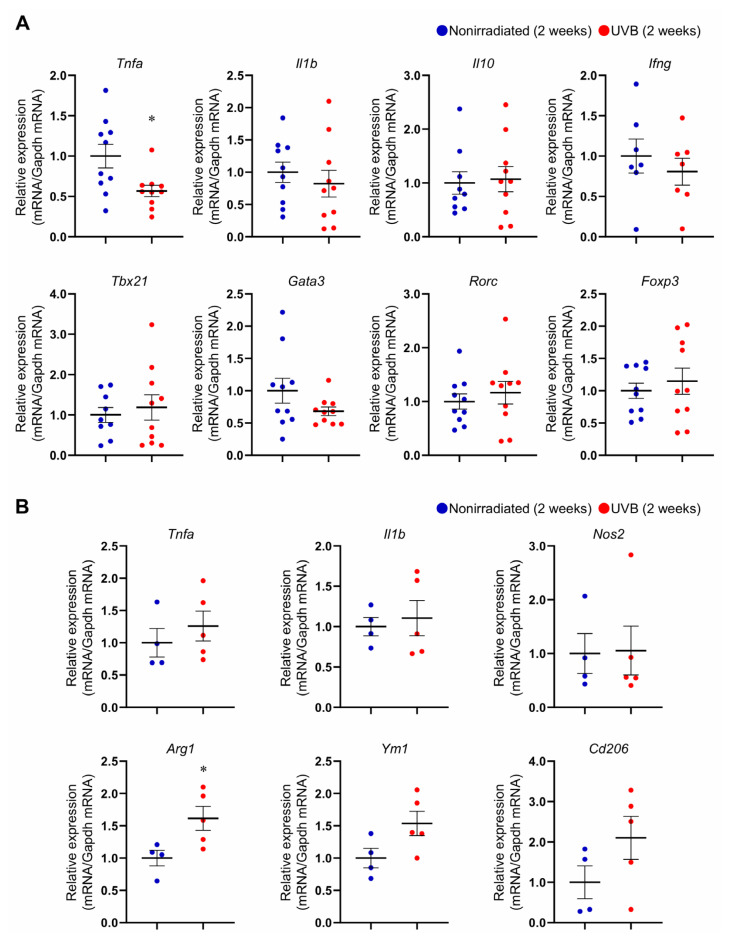
284 nm ultraviolet B (UVB) irradiation regulates aortic immunoinflammatory responses and induces a pro-resolving macrophage phenotype in mice with stabilized plaques **A**, Six- to eight-week-old male LDL receptor-deficient (*Ldlr*^−/−^) mice were fed a high-fat and high-cholesterol diet for 8 weeks to induce atherosclerotic lesions. At 14–16 weeks of age, the diet was changed to a standard diet, and the mice were irradiated with 284 nm UVB at 5 kJ/m^2^ twice weekly for 2 weeks. Thoracoabdominal aortas were harvested at 16–18 weeks of age. Nonirradiated male *Ldlr*^−/−^ mice served as controls. mRNA expression of pro-inflammatory cytokines (*Tnfa*, *Il1b*, and *Ifng*), anti-inflammatory cytokine (*Il10*), the helper T cell-related transcription factors (*Tbx21*, *Gata3*, and *Rorc*), and the Treg-specific transcription factor *Foxp3* in the aorta is shown. n = 7 to 10 per group. **B**, *Ldlr*^−/−^ mice were fed a high-fat and high-cholesterol diet for 8 weeks. At 14–16 weeks of age, the diet was changed to a standard diet, and the mice were irradiated with 284 nm UVB at 5 kJ/m^2^ twice weekly for 4 weeks. Three days after intraperitoneal injection of thioglycollate, peritoneal macrophages were isolated. mRNA expression of M1-type macrophage-related molecules (*Tnfa*, *Il1b*, and *Nos2*) and M2-type macrophage-related molecules (*Arg1*, *Ym1*, and *Cd206*) in peritoneal macrophages is shown. n = 4 to 5 per group. The expression levels of the target genes were normalized so that the mean values in nonirradiated mice were set to 1. GAPDH was used as an endogenous control reference. Data points represent individual animals. Horizontal bars represent means. Error bars indicate s.d. *P < 0.05; 2-tailed Student’s *t*-test: **B**; 2-tailed Welch’s *t*-test: **A**.

To determine whether 284 nm UVB irradiation regulates aortic immunoinflammatory responses, we examined the mRNA expression of inflammation-related molecules and transcription factors specific to helper T cell subsets and Tregs in the aorta by quantitative reverse transcription PCR. *Ldlr*^−/−^ mice were fed a high-fat and high-cholesterol diet for 8 weeks, and the diet was changed to a standard diet. We subsequently irradiated the mice twice weekly with 284 nm UVB for 2 weeks and harvested the thoracoabdominal aortas for quantitative reverse transcription PCR. The mRNA expression of the proinflammatory cytokine *Tnfa* was significantly downregulated in the aorta of UVB-irradiated mice, whereas there was no significant difference in the expression of other inflammation-related molecules, Th1-related *Tbx21*, Th2-related *Gata3*, Th17-related *Rorc*, or Treg-related *Foxp3*, between UVB-irradiated and nonirradiated mice (Fig. 4A).

Tregs regulate inflammatory immune responses and induce plaque regression partly by inducing the accumulation of pro-resolving M2-type macrophages in plaques (13), which indicates a common signature of stabilized and regressed plaques (20). We next investigated whether 284 nm UVB irradiation modulates the macrophage phenotype using peritoneal macrophages collected from the peritoneal cavity of mice with acute peritonitis. *Ldlr*^−/−^ mice were fed a high-fat and high-cholesterol diet for 8 weeks, after which the diet was changed to a standard diet. We subsequently irradiated the mice twice weekly with 284 nm UVB for 2 weeks and injected 3% thioglycollate to induce peritonitis. Three days after the injection, we isolated peritoneal macrophages and examined the mRNA expression of M1- or M2-type macrophage-related molecules by quantitative reverse transcription PCR. Interestingly, the mRNA expression of M2-type macrophage-related *Arg1* was markedly upregulated in the peritoneal macrophages isolated from UVB-irradiated mice (Fig. 4B). In addition, the mRNA expression of other M2-type macrophage-related molecules, such as *Ym1* and *Cd206*, tended to be increased following UVB irradiation (Fig. 4B). On the other hand, there was no significant difference in the expression of M1-type macrophage-related molecules between UVB-irradiated and nonirradiated mice (Fig. 4B).

Together, these results indicate that 284 nm UVB irradiation induces M2-type macrophage skewing, which may partly contribute to the promotion of inflammation resolution and stabilization of atherosclerotic plaques.

## DISCUSSION

Although recent intensive treatment reduces atherosclerotic disease events by modifying risk factors including lifestyle-related diseases, another important residual risk such as chronic inflammation should not be ignored (2). Accumulating clinical evidence indicates that the combination of conventional therapies and anti-inflammatory approaches can effectively prevent CAD in high-risk patients (3, 4). However, few effective therapies that directly modulate immunoinflammatory processes are available because anti-inflammatory therapies can cause severe infections derived from systemic immunosuppression or may not have sufficient ability to prevent fatal cardiac events. In addition, their high cost limits their clinical application. In the present study, using our unique UVB-LED device which we have recently developed, we demonstrated that in combination with lipid-lowering therapy, 284 nm UVB irradiation promotes the stabilization of established atherosclerotic plaques in the aortic sinus in hypercholesterolemic *Ldlr*^−/−^ mice without adverse effects. The plaque-stabilizing effect of UVB treatment was associated with augmented anti-inflammatory Treg immune responses in lymphoid tissues and the induction of a pro-resolving macrophage phenotype without affecting other immunity, which may indicate the low possibility of general immunosuppression. Considering that UVB-based phototherapy is inexpensive and an established therapy for cutaneous diseases with few adverse reactions (14), 284 nm UVB therapy could be a possible therapeutic approach for CAD.

Compelling experimental and clinical evidence suggests that adaptive immunity mediated by T cells is critically involved in the development and prevention of atherosclerosis, which may depend on the T cell subsets (1). CD4^+^Foxp3^+^ Tregs protect against atherosclerosis via modulation of immune cell functions, including a macrophage phenotype shift toward M2-type (13) and inhibition of Teff activation (21). Importantly, augmentation of Treg immune responses regulates the development of early and advanced atherosclerotic lesions and induces regression of established plaques (9–12, 15, 16). In this study, we found that 284 nm UVB irradiation expanded activated Tregs in the lymphoid tissues of hypercholesterolemic *Ldlr*^−/−^ mice under conditions of normalized plasma cholesterol levels, which could contribute to M2-type macrophage skewing and the stabilization of atherosclerotic plaques in the aortic root. These favorable effects of 284 nm UVB irradiation are in line with our recent study showing that this UVB treatment effectively prevented the development of atherosclerosis by augmenting Treg immune responses in hypercholesterolemic *Apoe*^−/−^ mice (16). Our finding of atherosclerotic lesion stabilization by the combination of 284 nm UVB irradiation and cholesterol normalization indicates that this UVB treatment may synergistically exert a plaque-stabilizing effect in combination with lipid-lowering therapy, which has important clinical implications.

As 284 nm UVB does not penetrate the epidermis, we suppose that 284 nm UVB irradiation would have a localized effect in the skin. Given our previous findings showing the critical role of skin Langerhans cells in broad-band UVB-dependent Treg functional modulation (15) and the possible involvement of increased proresolving lipid mediators in 284 nm UVB-dependent Treg expansion (16) in the skin or skin-draining LNs, we speculate that the localized effect of 284 nm UVB would exert a more dominant influence on Treg expansion than its systemic effect. UVB-expanded Tregs in the skin or skin-draining LNs may migrate to the spleen, leading to a modest increase in splenic Tregs. We believe that 284 nm UVB also exerts favorable systemic effects throughout the body by augmenting Treg responses.

Given that inflammatory conditions may disturb the suppressive function of Tregs (22), a combination approach involving the augmentation of Treg responses and reduction of inflammatory reactions could efficiently improve plaque stability. This is supported by our experimental study showing that combination therapy with anti-CD3 antibodies and IL-2 complexes effectively attenuated the development of atherosclerosis by augmenting Treg responses and suppressing Teff responses (11). Notably, our previous work reported that intravenous injection of anti-CD3 antibodies in combination with normalization of plasma cholesterol levels regressed and stabilized established atherosclerotic lesions by increasing the proportion of Tregs in lymphoid tissues and atherosclerotic lesions (10). Taken together with our finding that 284 nm UVB irradiation in combination with lipid-lowering therapy effectively augments Treg responses and limits inflammation, this strategy may be an effective approach for treating established atherosclerosis.

Based on the anti-inflammatory and immunomodulatory effects of UVB, UVB-based phototherapy is clinically used for treating various cutaneous diseases, with few major adverse reactions, if excessive exposure is avoided. Several wavelengths of UVB are well-validated and effective for treating cutaneous diseases (14), but the effect of 284 nm UVB irradiation on these diseases has not been examined. Given our previous and present data showing the anti-inflammatory and anti-atherogenic actions of 284 nm UVB irradiation (16), our UVB therapy could be an effective approach to atherosclerosis, although it should be carefully evaluated before its clinical application. In addition to the anti-atherogenic effect of 284 nm UVB irradiation, we reported the efficacy of 312 nm UVB irradiation for attenuating the development of atherosclerosis (17). These wavelengths of UVB have similar anti-inflammatory actions with some different mechanisms, whereas 284 nm UVB appears to limit the development of atherosclerosis and promote Treg responses more potently than 312 nm UVB (16). Despite this, as narrow-band UVB (a narrow peak around 311 nm) is clinically used for treating psoriasis with few adverse effects, it will be interesting to examine the plaque-stabilizing effect of similar 312 nm UVB in the context of clinical application. Our finding of the plaque-stabilizing action of 284 nm UVB irradiation is unique and interesting because we cannot receive this wavelength of UVB from natural sunlight due to interference with the atmosphere and the ozone layer, and no information on its biological effects has been provided. Given this background, it is important to discuss the safety of the clinical application of UVB therapy for atherosclerotic disease. The most significant adverse effect of UVB irradiation is carcinogenicity. Both 284 nm UVB and narrow-band UVB do not penetrate the epidermis, while the latter reaches deeper sites in the epidermis, potentially increasing the risk of cellular damage. When comparing 284 nm UVB with narrow-band UVB, the former is closer to the wavelength band where DNA absorbs energy, potentially posing a higher risk of direct DNA damage to cells. Despite these differences, no clear evidence exists for the relationship between UVB wavelength and cancer risk. To translate our experimental data to clinical settings, careful validation of the biological actions and safety of 284 nm UVB treatment is required.

In conclusion, we demonstrated that 284 nm UVB irradiation in combination with lipid-lowering therapy improves atherosclerotic plaque stability by augmenting anti-inflammatory Treg immune responses. We elucidated the unrecognized role of this specific wavelength of UVB in stabilizing atherosclerotic plaques. Our data suggest that the development of a novel 284 nm UVB phototherapy, which has not been clinically available and cannot be replaced by sunbathing, may provide an attractive strategy for treating high-risk patients with vulnerable atherosclerotic plaques.

## Figures and Tables

**Table I:** Antibodies for flow cytometry

Antibody	Clone	Fluorescent dye	Source	Catalog number
anti-CD4 Ab	RM4-5	PECy7	BD Biosciences	552775
anti-CD16/CD32 Ab	2.4G2	-	BD Biosciences	553142
anti-CD25 Ab	PC61	PE	BD Biosciences	553866
anti-CD44 Ab	IM7	PE	BD Biosciences	553134
anti-CD62L Ab	MEL-14	FITC	BD Biosciences	553150
anti-CD103 Ab	M290	FITC	BD Biosciences	557494
anti-CD152 Ab	UC10-4B9	PerCPCy5.5	BioLegend	106315
anti-Foxp3 Ab	FJK-16s	APC	Thermo Fisher Scientific	469457
anti-IFNγ	XMG1.2	PE	BD Biosciences	554412
anti-IL-4 Ab	11B11	PE	BD Biosciences	554435
anti-IL-10 Ab	JES5-16E3	APC	BD Biosciences	554468
anti-IL-17 Ab	TC11-18H10	APC	BD Biosciences	560184

**Table II:** Primer sequences for quantitative real-time reverse transcription PCR

Gene	Forward primer sequence (5′→3′)	Reverse primer sequence (5′→3′)
*Gapdh*	TGTGTCCGTCGTGGATCTGA	TTGCTGTTGAAGTCGCAGGAG
*Il1b*	TCCAGGATGAGGACATGAGCAC	GAACGTCACACACCAGCAGGTTA
*Il10*	GACCAGCTGGACAACATACTGCTAA	GATAAGGCTTGGCAACCCAAGTAA
*Tnfa*	CCACCACGCTCTTCTGTCTAC	AGGGTCTGGGCCATAGAACT
*Ifng*	CGGCACAGTCATTGAAAGCCTA	GTTGCTGATGGCCTGATTGTC
*Tbx21*	CTGCCTACCAGAACGCAGA	AAACGGCTGGGAACAGGA
*Gata3*	GGATGTAAGTCGAGGCCCAAG	ATTGCAAAGGTAGTGCCCGGTA
*Rorc*	CACAGAGACACCACCGGACAT	CGTGCAGGAGTAGGCCACATT
*Foxp3*	CTCATGATAGTGCCTGTGTCCTCAA	AGGGCCAGCATAGGTGCAAG
*Nos2*	GCAGAGATTGGAGGCCTTGTG	GGGTTGTTGCTGAACTTCCAGTC
*Arg1*	GGGAATCTGCATGGGCAAC	GCAAGCCAATGTACACGATGTC
*Ym1*	AGAAGGGAGTTTCAAACCTGGT	GTCTTGCTCATGTGTGTAAGTGA
*Cd206*	CAGGTGTGGGCTCAGGTAGT	TGTGGTGAGCTGAAAGGTGA

**Table III:** Body weight and plasma lipid profiles of baseline and UVB-irradiated or nonirradiated male *Ldlr*^−/−^ mice

	Baseline	Nonirradiated (2 weeks)	UVB (2 weeks)	Nonirradiated (4 weeks)	UVB (4 weeks)
Body weight (g) (n = 8–10)	20.3 ± 2.0	23.8 ± 1.1	24.3 ± 2.7	24.3 ± 1.1	24.2 ± 1.1
Total cholesterol (mg/dL) (n = 8–10)	1242.9 ± 133.6	474.7 ± 125.4###	391.0 ± 102.1###	270.3 ± 34.2#	224.6 ± 23.5###
HDL-cholesterol (mg/dL) (n = 8–10)	21.4 ± 6.9	62.7 ± 7.3###	63.9 ± 9.1###	65.4 ± 6.6###	65.6 ± 4.0###
Triglycerides (mg/dL) (n = 8–10)	45.5 ± 31.0	131.2 ± 63.9##	124.3 ± 31.6##	150.8 ± 49.1###	91.8 ± 32.9

All data are expressed as the mean ± s.d.

# *P* < 0.05,

## *P* < 0.01,

### *P* < 0.001 vs. baseline;

Kruskal-Wallis test followed by Dunn’s post hoc test: total cholesterol nonirradiated (4 weeks) and UVB (4 weeks) and triglycerides; 1-way ANOVA followed by Tukey’s multiple comparisons test: total cholesterol nonirradiated (2 weeks) and UVB (2 weeks) and HDL-cholesterol. *Ldlr*^−/−^ indicates LDL receptor-deficient; UVB, ultraviolet B; LDL, low-density lipoprotein; HDL, high-density lipoprotein.
